# Impact of hypoglycemia at the time of hospitalization for heart failure from emergency department on major adverse cardiovascular events in patients with and without type 2 diabetes

**DOI:** 10.1186/s12933-022-01651-0

**Published:** 2022-10-21

**Authors:** Seon-Ah Cha, Jae-Seung Yun, Gee-Hee Kim, Yu-Bae Ahn

**Affiliations:** 1 Division of Endocrinology & Metabolism, Department of Internal Medicine, College of Medicine, St. Vincent’s Hospital, The Catholic University of Korea, 93 Jungbu-daero, Paldal-gu, Seoul, Republic of Korea; 2 Division of Endocrinology and Metabolism, Department of Internal Medicine, Wonkwang University Sanbon Hospital, Gunpo, Republic of Korea; 3 Division of Cardiology, Department of Internal Medicine, College of Medicine, St. Vincent’s Hospital, The Catholic University of Korea, Seoul, Republic of Korea

**Keywords:** Hypoglycemia, Type 2 diabetes, Heart failure, MACE

## Abstract

**Background:**

Few studies have examined the association between hypoglycemic episodes among people with type 2 diabetes (T2DM) at the time of hospitalization for heart failure (HF) and cardiovascular outcomes.

**Methods:**

From March 2016 to June 2018, we conducted a retrospective cohort study to investigate hypoglycemia during HF hospitalization in the emergency department, three-point major adverse cardiovascular events (3P-MACE), and all-cause mortality; these were followed up through June 2021. HF hospitalization was defined according to American Heart Association criteria. Hypoglycemia was defined as a glucose level < 3.9 mmol/L at the time of HF hospitalization. We classified the enrolled patients into three groups (reference group, those without T2DM or hypoglycemia; those diagnosed with T2DM without hypoglycemia; and those with hypoglycemia and T2DM). We used Cox proportional hazard regression analysis to investigate the association between the three groups and the development of the first occurrence of 3P-MACE and all-cause mortality.

**Results:**

During a median of 25 months of follow-up, a total of 783 patients admitted due to HF were analyzed. In total, 159 (20.3%) cases of 3P-MACE were identified, and the mortality rate was 20.2% (n = 158). The median age of patients was 76.0 (65.0–82.0) years, and 49.0% were men. Patients with 3P-MACE had a lower body mass index (22.6 [20.4–25.1] vs. 23.8 [21.3–26.7]), higher frequency of previous history of HF (24.5% vs. 15.7%), T2DM (64.2% vs. 47.3%), higher rates of hypoglycemia at the time of HF hospitalization (19.5% vs. 7.7%), and lower eGFR levels (61.1 [36.0–80.7] mL/min/1.73 m^2^ vs. 69.2 [45.8–89.5] mL/min/1.73 m^2^) than those without 3P-MACE. The multivariable adjusted HR of 3P-MACE was as follows: group with hypoglycemia and T2DM: HR, 2.29; 95% CI: 1.04–5.06; group with T2DM without hypoglycemia: HR: 1.42; 95% CI: 0.86–2.33; and all-cause mortality group with hypoglycemia and T2DM: HR: 2.58; 95% CI: 1.26–5.31, group with T2DM without hypoglycemia: HR: 1.32; 95% CI: 0.81–2.16; compared to the reference group (group without T2DM or hypoglycemia).

**Conclusions:**

T2DM and hypoglycemia are independent risk factors for 3P-MACE and all-cause mortality compared to those without hypoglycemia during HF hospitalization.

**Supplementary Information:**

The online version contains supplementary material available at 10.1186/s12933-022-01651-0.

## Background

In 2019, 9.3% of people aged 20–79 years had type 1 or type 2 diabetes (T2DM) worldwide [1]. In addition, there will be 578 million (10.2%) adults diagnosed with type 1 diabetes or T2DM by 2030. This increase in T2DM has been associated with a higher incidence of micro- and macrovascular complications [1].

Tight glycemic control showed beneficial effects on microvascular complications but inconsistent results on macrovascular complications in patients with T2DM. Strict glycemic control is inevitably associated with an increased risk of hypoglycemia and severe hypoglycemia in T2DM patients [2]. Severe hypoglycemia is associated with unexpected and recurrent morbidity in patients with type 1 diabetes and T2DM and is occasionally fatal [3]. Several studies and post hoc analyses of the Veterans Affairs Diabetes Trial showed that severe hypoglycemia was linked to increased CV disease [4, 5, 5]. Action to Control Cardiovascular Risk in Diabetes (ACCORD) study participants also showed that severe hypoglycemia episodes during a 24 month period have a 1.68-fold higher risk of incident HF than those without any episode of hypoglycemia [6]. In addition, a dose‒response relationship was observed between severe hypoglycemic episodes and myocardial infarction, stroke, heart failure, and all-cause death in the Korean population with T2DM [7]. Accordingly, current clinical guidelines emphasize that patients with T2DM should be checked for the occurrence and risk of hypoglycemia at every visit [8].

Previous studies reported that 10–47% of patients with HF had T2DM, while T2DM was associated with a threefold risk of HF compared to those without T2DM [9–11]. Moreover, patients with either type 1 or type 2 diabetes and HF have a 1.7-fold increased risk of myocardial infarction (MI), stroke, or cardiovascular (CV) death at 4 years compared to patients without type 1 diabetes or T2DM [12].

A decreasing trend in major CV complications was observed from 2006 to 2013 in Korea; these complications included hospitalization due to ischemic heart disease (− 29.5% vs. − 14.7%), MI rate (− 37.3% vs. − 25.5%), and ischemic stroke rate (− 37.0% vs. − 28.9%) [13]. However, the prevalence of HF has been increasing in T2DM patients (men, 72–146 per 10,000 adults, women, 124–161 per 10,000 adults) from 2006 to 2015 in Korea [13]. Despite complex medical therapies addressing the underlying causes of HF, including ischemic heart disease, hyperglycemia, dyslipidemia, and hypertension, these patients had substantially higher mortality with T2DM and HF than without T2DM, emphasizing the need to estimate residual risk factors for mortality in T2DM and HF [4, 13].

However, long-term data on the effect of hypoglycemia on adverse CV outcomes in HF hospitalization are limited. Conducting glycemic control research in patients with HF and T2DM is crucial for understanding the target blood glucose level for their treatment. Therefore, the objective of this study was to estimate the CV outcomes and all-cause mortality associated with hypoglycemia and T2DM in patients at the time of HF hospitalization.

## Method

### Study design and oversight

We performed retrospective cohort study of 783 patients aged ≥ 25 years who were admitted to St. Vincent’s Hospital in South Korea for HF via the emergency department between March 2016 and June 2018, consecutively followed up until June 2021. Patients with gestational diabetes, type 1 diabetes, thyroid disease, and severe illnesses, including liver cirrhosis, malignancy, or sepsis, were excluded from the current investigation. All study protocols were approved by the Institutional Review Board of the Catholic Medical Center Ethics Committee (VC20RISI0253).

### Measurements

All participants were interviewed about their medical history and their anthropometric measurements were recorded, as described in prior publications [13]. In brief, information on medical history, medication, and current or past smoking status was obtained [14]. Fasting plasma glucose and lipid profiles were assessed using an automated enzymatic method (736–40; Hitachi, Tokyo, Japan) after 8 h of fasting, and HbA1c was measured using high-performance liquid chromatography (Bio-Rad, Montreal, QC, Canada). The estimated glomerular filtration rate (eGFR) was estimated using the four-component Modification of Diet in Renal Disease equation.

The N-terminal-pro-B-type natriuretic peptide (NT-proBNP) level was measured using an electrochemiluminescence sandwich immunoassay method with an Elecsys 2010 analyzer (Roche Diagnostics, Basel, Switzerland), and high-sensitivity troponin T and creatinine kinase MB isoenzyme were determined using the same method with an autoanalyzer (Cobas e 411, Roche Diagnostics) on the day of admission.

All participants had transthoracic echocardiographic data within three days of hospital admission to assess the indices of cardiac function and structure. Transthoracic echocardiography was performed using a Vivid Seven ultrasound machine (GE Medical Systems, Horten, Norway) to estimate morphologic and hemodynamic parameters with a 2.5 MHz transducer. Standard two-dimensional measurements, including left ventricular diastolic and systolic dimensions, ventricular septum and posterior wall thickness, and left atrial volume, were obtained as recommended by the American Society of Echocardiography [15].

### Definition

Hypoglycemia was defined as “blood glucose < 3.9 mmol/L” and checked at the time of admission to the hospital for HF from the emergency department. Accordingly, we classified the enrolled patients into three groups (those without T2DM or hypoglycemia; those diagnosed with T2DM without hypoglycemia; and those with hypoglycemia and T2DM).

HF hospitalization was defined as an event that met all of the following criteria by the American Heart Association [16]. The patient (i) was admitted to the hospital with a primary diagnosis of HF, (ii) was hospitalized for at least 24 h, (iii) exhibited documented new or worsening symptoms due to HF on presentation, (iv) had objective evidence of new or worsening HF, and (v) received or intensified at least one treatment specifically for HF [16]. HF hospitalization included first-time HF hospitalization or rehospitalization.

The patient was considered to have T2DM if they were treated with T2DM medication or lifestyle modification at baseline or if they had at least two fasting plasma glucose measurements ≥ 7 mmol/L or HbA1c of ≥ 6.5%.

CV death includes death resulting from an acute MI within 30 days after initial MI, death due to HF, sudden cardiac death, death due to stroke, death due to CV procedures, death due to CV hemorrhage, and death due to other CV causes [16].

The term MI was used when there was evidence of myocardial necrosis in a clinical setting consistent with myocardial ischemia [16]. The diagnosis of MI required a combination of evidence of myocardial necrosis, including cardiac biomarkers and additional information from the clinical presentation, electrocardiographic changes, or the results of coronary artery imaging.

Stroke was defined as an acute episode of focal or global neurological dysfunction caused in the brain as a result of hemorrhage or infarction [16]. Chronic kidney disease (CKD) was defined as at least two measures of eGFR < 60 mL/min/1.73 m^2^ over 3 months or more [17].

### Primary and secondary outcomes

The primary outcome was the first occurrence of three-point major adverse cardiovascular events (3P-MACE), a composite of death from CV causes, nonfatal MI, or nonfatal stroke. Secondary outcomes included all-cause mortality.

If a subject experienced more than one event, the first event was considered in the analysis.

### Statistical analysis

All data are expressed as the mean ± standard deviation for normal distribution, median and interquartile range for nonnormal distribution, or frequency. Continuous symmetrical variables were tested using an independent *t test*, asymmetrical variables were tested using the Mann‒Whitney test, and the chi-square test was used for categorical variables.

A Cox proportional hazard regression model was used for the associations between the three groups, 3P-MACE, and all-cause mortality with prespecified covariates of age, sex, body mass index, history of cardiovascular disease (CVD), HF, etiology of HF, presence of CKD, systolic blood pressure (SBP), fasting plasma glucose, HbA1c, use of insulin, sulfonylurea, metformin, antihypertensive medications including angiotensin-converting enzyme inhibitors/angiotensin receptor blockers, β-blockers, and diuretics including mineralocorticoid receptor antagonists, aspirin, statin, troponin-T, NT-proBNP, C-reactive protein, and reduced ejection fraction (EF ≤ 40%). The proportional hazards assumption was estimated using a time interaction term with survival time in the regression model and log–log survival plots. There was no significant departure from proportionality to hazards over time. Potential confounders were identified a priori based on a literature review. Statistical significance was evaluated using two-sided tests, with the level of significance set at 0.05. Statistical analyses were performed using SAS version 9.3 (SAS Institute, Cary, NC, USA).

## Results

### Study outline

**Fig. 1:**
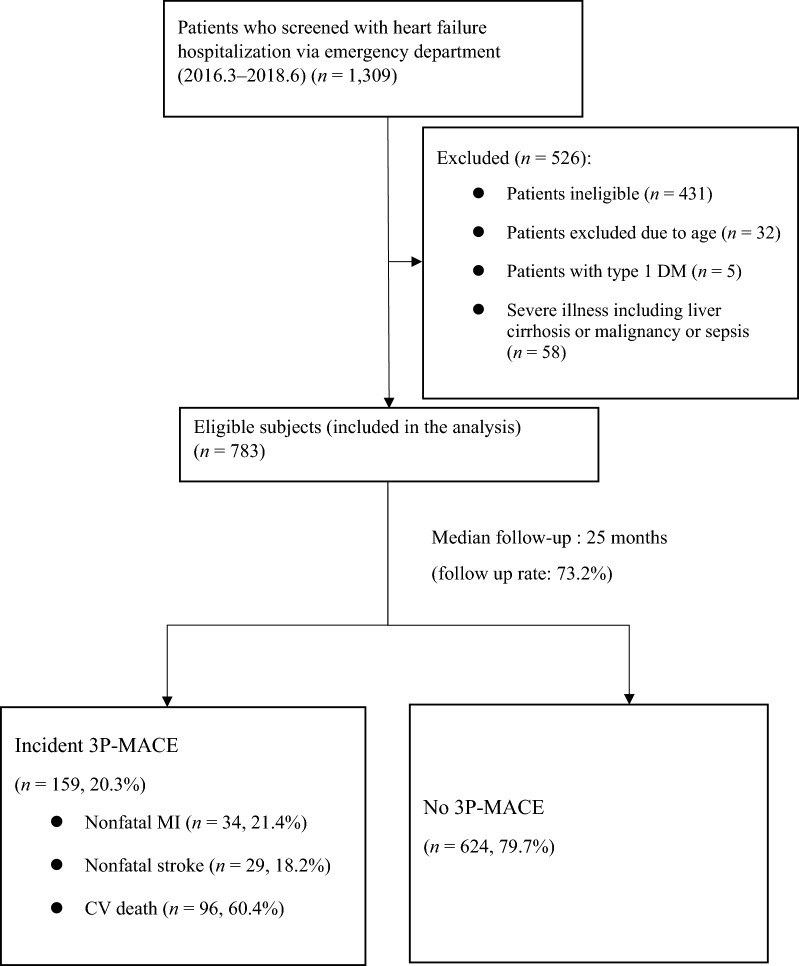
Study outline. 3P-MACE three-point major adverse cardiovascular events

A total of 1309 subjects admitted to St. Vincent’s Hospital due to HF were consecutively screened; 526 subjects were excluded: 431 patients who were misclassified, 32 excluded due to age, 5 with T1DM, and 58 with severe illness, including liver cirrhosis, malignancy, or sepsis. Finally, 783 patients admitted for HF hospitalization from the emergency department were recruited between March 2016 and June 2018 (Fig. 1). A total of 158 (20.2%) patients died, and 159 patients (20.3%) underwent 3P-MACE during the median follow-up period (interquartile range, 7.0–35.0 months).

### Baseline characteristics

The median age of the patients was 76.0 (interquartile range, 65.0–82.0) years, and 49.0% were men. The mean left ventricular ejection fraction was 41.9 ± 15.4%. A total of 397 patients (50.7%) had T2DM. The etiology of HF was ischemic heart disease (29.2%) or nonischemic heart disease (70.8%) (Table 2). The median hospital stay was 6.0 days (interquartile range, 4.0–10.0 days).

A total of 386 patients (49.3%) were in the reference group (HF without T2DM or hypoglycemia group), 318 (40.6%) were in the group with T2DM without hypoglycemia, and 79 (10.1%) were in the group with T2DM and hypoglycemia.

Several baseline characteristics were significantly different between the groups. As shown in Table 1, fasting plasma glucose (5.7 ± 0.8 mmol/L, 8.5 ± 4.1, and 3.3 ± 0.7 mmol/L, p < 0.001), HbA1c level (5.7 ± 0.5%, 7.3 ± 3.3%, and 7.8 ± 1.8%, p < 0.001), eGFR (74.8 [56.7–99.6] mL/min/1.73 m^2^, 57.1 [36.5–83.8] mL/min/1.73 m^2^, and 45.9 [29.4–69.2] mL/min/1.73 m^2^, p < 0.001) showed significant differences among the three groups. The group with T2DM and hypoglycemia had a reduced left ventricular ejection fraction (38.7 ± 14.6% vs. 43.5 ± 15.0%, p = 0.029) compared with those with T2DM without hypoglycemia (Table 1).

**Table 1:** Baseline characteristics of patients according to the presence of type 2 diabetes or hypoglycemia with heart failure hospitalization

	Heart failure without T2 DM	Heart failure with T2DM without hypoglycemia	Heart failure with T2DM, hypoglycemia	*P* value
*n*	386 (49.3)	318 (40.6)	79 (10.1)	
Age (years)	71.6 ± 15.6	73.2 ± 11.3	72.6 ± 13.7	0.269
Male	196 (50.8)	147 (46.2)	41 (51.9)	0.421
Body mass index (kg/m^2^)	22.8 (20.4–26.5)	24.0 (22.1–26.6)	23.5 (20.8–26.3)	0.002
Smoking				*0.095*
Current	66 (17.1)	48 (15.1)	8 (10.1)	
Ex-smoker	30 (7.8)	36 (11.3)	13 (16.4)	
T2DM	0 (0.0)	318 (100.0)	79 (100.0)	< 0.001
Duration of T2DM (years)	-	11.2 ± 10.4	14.3 ± 11.7	0.044
Hypertension	213 (55.2)	239 (75.2)	62 (78.5)	< 0.001
History of CHD	45 (11.7)	71 (22.3)	24 (30.4)	< 0.001
History of stroke	33 (8.5)	35 (11.0)	9 (11.4)	0.490
Previous HF	59 (15.3)	58 (18.2)	20 (25.3)	0.092
Duration of HF (years)	3.2 ± 2.8	6.1 ± 5.3	3.8 ± 3.1	0.026
Etiology of HF				* < 0.001*
Ischemic cause (coronary artery disease or myocardial infarction)	85 (22.0)	110 (34.6)	34 (43.0)	
Nonischemic cause	301 (78.0)	208 (65.4)	45 (57.0)	
Chronic kidney disease	107 (27.7)	166 (52.2)	54 (68.4)	< 0.001
Systolic blood pressure (mm Hg)	129.0 ± 26.2	135.0 ± 28.8	135.1 ± 26.4	0.010
Diastolic blood pressure (mm Hg)	77.9 ± 15.8	80.3 ± 16.7	77.8 ± 13.6	0.126
Heart rate (beats per min)	92.5 ± 23.7	92.8 ± 21.9	93.7 ± 23.3	0.910
Left ventricular ejection fraction (%)	41.3 ± 15.9	43.5 ± 15.0	38.7 ± 14.6	0.029
E/e’	19.0 ± 9.5	21.0 ± 10.2	20.9 ± 9.0	0.020
Laboratory variables				
FPG (mmol/L)	5.7 ± 0.8	8.5 ± 4.1	3.3 ± 0.7	< 0.001
HbA1c (%)	5.7 ± 0.5	7.3 ± 3.3	7.8 ± 1.8	< 0.001
eGFR (mL min^−1^ 1.73 m^−2^)	74.8 (56.7–99.6)	57.1 (36.5–83.8)	45.9 (29.4–69.2)	< 0.001
Total cholesterol (mmol/L)	3.9 ± 1.0	3.8 ± 1.1	3.5 ± 1.8	0.072
Triglyceride (mmol/L)	0.3 (0.7–1.1)	1.0 (0.8–1.4)	0.8 (0.7–1.1)	< 0.001
HDL-C (mmol/L)	1.1 ± 0.3	1.1 ± 0.4	1.1 ± 0.3	0.140
LDL-C (mmol/L)	2.4 ± 0.8	2.4 ± 0.9	2.2 ± 0.9	0.105
NT-pro BNP (pg/mL)	3900 (1707–7928)	4250 (1828–9598)	7485 (3266–24,153)	< 0.001
hsTnT (ng/L)	25.0 (16.0–50.5)	32.0 (18.0–75.5)	66.0 (27.0–133.3)	< 0.001
CK-MB (ng/ml)	3.2 (2.1–5.6)	3.1 (2.1–5.5)	4.1 (2.4–6.3)	0.104
CPK (U/L)	104.0 (69.0–174.5)	103.0 (66.0–166.5)	104.0 (62.0–164.0)	0.722
CRP (mg/dl)	0.5 (0.2–1.7)	0.5 (0.2–2.1)	1.0 (0.2–3.2)	0.132
Medication				
Cardiovascular medication				
ACEi/ARB	115 (29.8)	122 (38.4)	29 (36.7)	0.050
ß-blocker	84 (21.8)	98 (30.9)	25 (31.6)	0.013
CCB	60 (15.5)	96 (30.4)	28 (35.4)	< 0.001
Diuretics	148 (38.3)	143 (45.0)	45 (57.0)	0.006
Aspirin	77 (19.9)	87 (27.4)	26 (32.9)	0.012
Statin	68 (17.6)	106 (33.3)	29 (36.7)	< 0.001
Diabetes treatment				
Insulin	0 (0.0)	29 (9.1)	11 (13.9)	0.204
Sulfonylurea	0 (0.0)	59 (18.6)^#^	30 (38.0)^#^	< 0.001
Metformin	0 (0.0)	94 (29.6)	26 (32.9)	0.562
DPP-4 inhibitor	0 (0.0)	99 (31.1)	28 (35.4)	0.462

Data are number (percentage) or medians with 25th–75th percentiles, means ± SD. *P* < 0.05 was considered significant

*CHD* coronary heart disease, *T2DM* type 2 diabetes, *FPG* fasting plasma glucose, *eGFR* estimated glomerular filtration rate, *HDL* high-density lipoprotein, *LDL* low-density lipoprotein, *NT-proBNP* N-terminal-pro-B-type natriuretic peptide, hsTnT High-sensitivity troponin T, *CRP* C-reactive protein, *ACEi/ARB* ACE inhibitor/angiotensin receptor blocker, *CCB* calcium channel blocker

^#^*P* < 0.05 (comparison between heart failure with T2DM group and heart failure with T2DM and hypoglycemia)

During a median follow-up period of 25 months, 159 patients (20.3%) developed 3P-MACE. Compared to those without 3P-MACE, patients with 3P-MACE had a lower body mass index (22.6 [20.4–25.1] kg/m^2^ vs. 23.8 [21.3–26.7] kg/m^2^, p = 0.002), a higher frequency of history of T2DM (64.2% vs. 47.3%, p < 0.001), hypoglycemia at HF hospitalization (19.5% vs. 7.7%, p < 0.001), previous diagnosis of HF (24.5% vs. 15.7%, p = 0.009), CKD (49.7% vs. 39.7%, p = 0.023), and higher high-sensitivity troponin T levels (54.0 [27.0–122.8] ng/L vs. 26.0 [16.0–55.5] ng/L, p < 0.001), NT-pro-BNP levels (7490 [3002–19361] pg/mL vs. 3825 [1603–7922] pg/mL, p < 0.001) than patients without 3P-MACE (Table 2).

**Table 2:** Baseline characteristics according to the development of incident three-point major adverse cardiovascular events (3P-MACE) in patients with heart failure hospitalization

	Total (n = 783)	3P MACE (-) (n = 624)	3P MACE ( +) (n = 159)	*P* value
Age (years)	72.3 ± 13.8	72.4 ± 13.9	71.7 ± 13.8	0.472
Male	384 (49.0)	297 (47.6)	87 (54.7)	0.109
Body mass index (kg/m^2^)	23.5 (21.2–26.5)	23.8 (21.3–26.7)	22.6 (20.4–25.1)	0.002
Smoking (current)	122 (15.6)	95 (15.2)	27 (17.0)	0.390
T2DM	397 (50.7)	295 (47.3)	102 (64.2)	< 0.001
Duration of T2DM (years)	12.0 ± 10.8	12.2 ± 10.9	11.2 ± 10.7	0.484
Hypertension	514 (65.6)	402 (64.4)	112 (70.4)	0.154
History of CHD	140 (17.9)	101 (16.2)	39 (24.5)	0.014
History of stroke	77 (9.8)	60 (9.6)	17 (10.7)	0.684
History of heart failure	137 (17.5)	98 (15.7)	39 (24.5)	0.009
Duration of HF (years)	4.6 ± 4.4	5.3 ± 5.0	3.6 ± 2.9	0.206
Etiology of heart failure				*0.002*
Ischemic cause (coronary artery disease or myocardial infarction)	229 (29.2)	167 (26.8)	62 (39.0)	
Nonischemic cause	554 (70.8)	457 (73.2)	97 (61.0)	
Chronic kidney disease	317 (41.8)	248 (39.7)	79 (49.7)	0.023
Systolic blood pressure (mm Hg)	132.0 ± 27.4	133.1 ± 27.4	128.1 ± 27.5	0.101
Diastolic blood pressure (mm Hg)	79.0 ± 16.0	79.5 ± 15.9	76.4 ± 16.2	0.056
Heart rate (beats per min)	92.7 ± 23.0	92.6 ± 23.3	93.1 ± 21.8	0.397
Left ventricular ejection fraction (%)	41.9 ± 15.4	43.2 ± 15.5	36.8 ± 14.1	< 0.001
E/e’	19.9 ± 9.7	19.3 ± 9.2	23.0 ± 11.2	< 0.001
Hypoglycemia during hospitalization	79 (10.1)	48 (7.7)	31 (19.5)	< 0.001
Laboratory variables				
FPG (mmol/L)	6.6 ± 3.2	6.6 ± 3.0	6.9 ± 4.0	0.754
HbA1c (%)	6.7 ± 2.5	6.7 ± 2.7	7.0 ± 1.6	0.002
eGFR (mL min^−1^ 1.73 m^−2^)	66.8 (45.0–87.7)	69.2 (45.8–89.5)	61.1 (36.0–80.7)	0.003
Total cholesterol (mmol/L)	3.8 ± 1.1	3.8 ± 1.0	3.8 ± 1.1	0.916
Triglyceride (mmol/L)	0.9 (0.7–1.2)	0.9 (0.7–1.2)	0.9 (0.7–1.1)	0.962
HDL-C (mmol/L)	1.1 ± 0.3	1.1 ± 0.3	1.1 ± 0.3	0.456
LDL-C (mmol/L)	2.4 ± 0.9	2.4 ± 0.8	2.5 ± 0.9	0.533
NT-pro BNP (pg/mL)	4238 (1860–9302)	3825 (1603–7922)	7490 (3002–19,361)	< 0.001
hsTnT (ng/L)	29.0 (17.0–65.0)	26.0 (16.0–55.5)	54.0 (27.0–122.8)	< 0.001
CK-MB (ng/ml)	3.2 (2.1–5.6)	3.2 (2.1–5.4)	3.7 (2.2–6.8)	0.044
CPK (U/L)	104.0 (68.5–170.0)	103.0 (69.0–167.3)	105.0 (65.0–191.0)	0.891
CRP (mg/dl)	0.5 (0.2–2.0)	0.5 (0.2–1.8)	0.8 (0.3–2.9)	0.010
Medication			***-***	
Cardiovascular medication				
ACEi/ARB	266 (34.0)	214 (34.3)	52 (32.7)	0.705
ß-blocker	207 (26.5)	170 (27.3)	37 (23.3)	0.305
CCB	184 (23.6)	149 (24.0)	35 (22.0)	0.607
Diuretics	336 (42.9)	252 (40.4)	84 (52.8)	0.005
Aspirin	190 (24.3)	148 (23.7)	42 (26.4)	0.479
Statin	203 (25.9)	162 (26.0)	41 (25.8)	0.964
Diabetes treatment				
Insulin	40 (5.1)	32 (5.1)	8 (5.0)	0.961
Sulfonylurea	89 (11.4)	63 (10.1)	26 (16.4)	0.027
Metformin	120 (15.3)	88 (14.1)	32 (20.1)	0.060
DPP-4 inhibitor	127 (16.2)	94 (15.1)	33 (20.8)	0.082

Data are number (percentage) or medians with 25th–75th percentiles, means ± SD. *P* < 0.05 was considered significant

*CHD* coronary heart disease, *T2DM*, type 2 diabetes, *FPG* fasting plasma glucose, *eGFR* estimated glomerular filtration rate, *HDL* high-density lipoprotein, *LDL* low-density lipoprotein, *NT-proBNP* N-terminal-pro-B-type natriuretic peptide, *hsTnT* High-sensitivity troponin T, *CRP* C-reactive protein, *ACEi/ARB* ACE inhibitor/angiotensin receptor blocker, *CCB* calcium channel blocker

In addition, there was a higher use of sulfonylurea (16.4% vs. 10.1%) in patients with 3P-MACE than in those without 3P-MACE.

The median time from HF hospitalization to 3P-MACE was 7 months (interquartile range 1.0–19.0 months). Of the 159 patients who developed 3P-MACE, 31 (39.2%) were in the group with T2DM and hypoglycemia, 71 (22.3%) were in the group with T2DM without hypoglycemia, and 57 (14.8%) were in the reference group (those without T2DM or hypoglycemia) (p for trend < 0.001).

### Relationship between type 2 diabetes, hypoglycemia, 3P-MACE, and all-cause mortality

Table 3 and Additional file 1: Table S3 show that the presence of T2DM and hypoglycemia (blood glucose ≤ 3.9 mmol/L) at the time of HF hospitalization was a significant risk factor for 3P-MACE.

**Table 3:** Increased risk for cardiovascular events and all-cause mortality according to presence of type 2 diabetes or hypoglycemia in heart failure hospitalization

	Patients without T2DM	Patients with T2DM without hypoglycemia	Patients with T2DM and hypoglycemia
	HR (95% CI)	HR (95% CI)	HR (95% CI)
3P-MACE			
No. of cases (%)	57 (14.8)	71 (22.3)	31 (39.2)
Model 1^a^	1 [Reference]	1.39 (0.98–1.97)	2.67 (1.72–4.15)
Model 2^b^	1 [Reference]	1.64 (1.06–2.55)	3.53 (1.92–6.49)
Model 3^c^	1 [Reference]	1.42 (0.86–2.33)	2.29 (1.04–5.06)
Cardiovascular mortality			
No. of cases (%)	37 (9.6)	37 (11.6)	22 (27.8)
Model 1^a^	1 [Reference]	1.14 (0.72–1.81)	3.28 (1.93–5.56)
Model 2^b^	1 [Reference]	1.46 (0.83–2.56)	4.95 (2.35–10.42)
Model 3^c^	1 [Reference]	1.12 (0.61–2.07)	2.87 (1.17–7.05)
All-cause mortality			
No. of cases (%)	55 (14.2)	69 (21.7)	34 (43.0)
Model 1^a^	1 [Reference]	1.42 (1.00–2.02)	3.37 (2.15–5.19)
Model 2^b^	1 [Reference]	1.74 (1.13–2.69)	4.53 (2.52–8.16)
Model 3^c^	1 [Reference]	1.32 (0.81–2.16)	2.58 (1.26–5.31)

HR, hazard ratio, CI confidence interval, T2DM, type 2 diabetes

^a^Model 1 was adjusted for age and sex

^b^Model 2 additionally included body mass index, current smoking status, presence of previous coronary heart disease, stroke, heart failure, etiology of heart failure, duration of diabetes (if subjects had T2DM), systolic blood pressure, fasting plasma glucose, HbA1c ≥ 7%, and presence of chronic kidney disease (eGFR ≤ 60 mL/min/1.73 m^2^)

^c^Model 3 additionally included use of antihypertensive medications, statins, insulin, sulfonylurea, high-sensitivity troponin T, N-terminal-pro-B-type natriuretic peptide, C-reactive protein level, and reduced EF (EF ≤ 40%)

In addition, the impact of hypoglycemia on 3P-MACE was substantially greater (HR: 2.29; 95% CI: 1.04–5.06) than that of the reference group (those without T2DM or hypoglycemia) after adjusting for age, sex, body mass index, diabetes duration (≥ 10 years), history of CVD, HF, etiology of HF, presence of CKD, SBP, fasting plasma glucose, HbA1c, use of insulin, sulfonylurea, antihypertensive medication, statin, levels of high-sensitivity troponin T, NT-proBNP, and C-reactive protein. In terms of the T2DM without hypoglycemia group, the HR was greater than that of the reference group (those without T2DM or hypoglycemia), but the difference was not significant.

Moreover, body mass index (p = 0.006), history of HF (P = 0.008), SBP (p = 0.001), and NT-proBNP level (p = 0.010) were also independent covariates for 3P-MACE in HF patients in this study (Additional file 1: Table S3).

There were no significant interactions between the effect of hypoglycemia and body mass index (p-interaction = 0.866), SBP (p-interaction = 0.243), use of sulfonylurea (p-interaction = 0.101), use of insulin (p-interaction = 0.934), and NT-proBNP (p-interaction = 0.810) on the risk of 3P-MACE. A significant interaction of the effect of hypoglycemia and previous HF with the risk of 3P MACE was observed in this study (p for interaction = 0.036).

**Fig. 2:**
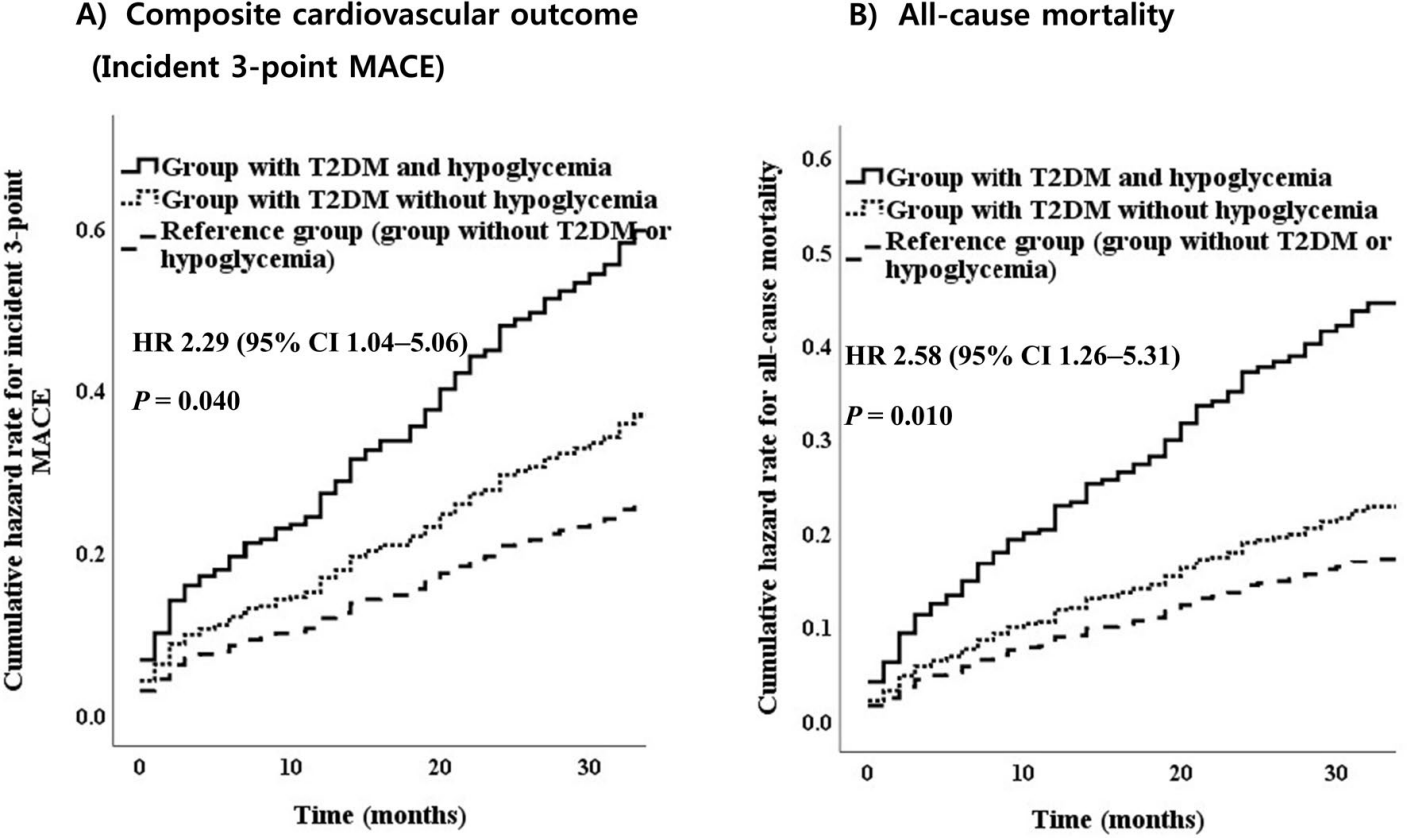
Cumulative hazard rate of the development of cardiovascular outcome and all-cause mortality according to the presence of T2DM and hypoglycemia in patients with heart failure hospitalized in the emergency department. HR; hazard ratio, T2DM; type 2 diabetes

The cumulative hazard rate (HR) of the development of incident 3P MACE and all-cause mortality according to T2DM or hypoglycemia is shown in Fig. 2. The highest rate of all-cause mortality was noted in the group with T2DM and hypoglycemia. Thirty-three patients (42.3%) in the group with T2DM and hypoglycemia, 70 patients (21.9%) in the group with T2DM without hypoglycemia, and 55 patients (14.2%) in the reference group (those without T2DM or hypoglycemia) died, and the HR of the group with T2DM and hypoglycemia for all-cause mortality was 2.58 (95% CI: 1.26–5.31) (p for trend = 0.002).

The mortality rate at 1 year was 12.7% in all patients with HF, 10.1% in the reference group, 11.0% in the group with T2DM without hypoglycemia, and 32.9% in the group with T2DM and hypoglycemia. The mortality rate at 2 years was 17.6% (138) in patients with HF, 13.5% in the reference group (those without T2DM or hypoglycemia), 17.0% in the group with T2DM without hypoglycemia, and 40.5% in the group with T2DM and hypoglycemia.

Older age (p = 0.022), lower body mass index (p < 0.001), history of HF (p = 0.023), SBP (p = 0.012), high-sensitivity troponin T (p = 0.025), and NT-proBNP (p = 0.002) were also significant predictors of all-cause mortality. However, there were no significant interactions between the effects of hypoglycemia and age (p-interaction = 0.810), body mass index (p-interaction = 0.387), SBP (p-interaction = 0.703), high-sensitivity troponin T (p-interaction = 0.423), and NT-proBNP (p-interaction = 0.685) on the risk of all-cause mortality.

A similar result was found for CV mortality (Table 3): T2DM and hypoglycemia were a significant predictors of CV mortality (HR: 2.87; 95% CI: 1.17–7.05, group with T2DM without hypoglycemia; HR: 1.12; 95% CI: 0.61–2.07) compared to the reference group (those without T2DM or hypoglycemia).

## Discussion

This study found that hypoglycemia and T2DM were associated with increased overall mortality and adverse CV outcomes compared to patients without T2DM during HF hospitalization from the emergency department, even after adjusting for multiple covariates. Covariates for CV outcome and all-cause mortality included age, sex, body mass index, diabetes duration, smoking status, history of CVD, history and etiology of HF, SBP, fasting plasma glucose, HbA1c, CKD (eGFR ≤ 60 mL/min/1.73 m^2^), presence of systolic HF (EF ≤ 40%), use of antihypertensive drugs, insulin, sulfonylurea, statins, high-sensitivity troponin T, NT-proBNP, and C-reactive protein levels. Among the components of 3P-MACE, the presence of T2DM and hypoglycemia has a greater association with the development of CV mortality and nonfatal MI. To our knowledge, this study is one of the first to investigate the effect of hypoglycemia in HF hospitalization on 3P-MACE and all-cause mortality.

### Type 2 DM in heart failure

Previous studies have shown that chronic hyperglycemia is associated with the risk of HF in T2DM, with a 1.15-fold risk for HF proportional to an increase of 1% in HbA1c [18]. HF is related to increased mortality and CV mortality; in particular, the clinical outcome appears worse in T2DM and even in prediabetes [19]. T2DM is related to significant changes in myocardial structure and function via overt myocardial ischemia and an increased risk of HF, the so-called cardiovascular disease continuum [20–22]. T2DM is also associated with higher levels of atherogenic dyslipidemia, which leads to thrombosis, inflammation, plaque ulceration, accumulation of advanced glycation end-products that cross-link extracellular matrix proteins and transduce fibrogenic signals, endothelial dysfunction, and oxidative stress [9, 23].

### Hypoglycemia in heart disease, heart failure

The combination of hypoglycemia and T2DM had a stronger negative impact on 3P-MACE and all-cause mortality among HF hospitalizations than T2DM without hypoglycemia in this study. Aguilar et al. showed that the association between baseline HbA1c in T2DM and HF and all-cause mortality appears U-shaped, with the lowest risk of all-cause mortality at HbA1c of 7.1–7.8% [24]. A similar result was shown in a general HF population in a UK study [25]. In addition, an observational cohort study showed a U-shaped relationship between time-weighted mean HbA1c and mortality, which showed the lowest risk in patients with HbA1c of 7.1–8.0%, and the U-shape is present in drug-treated but not in diet-treated T2DM [26]. This result corresponds to the Action to Control Cardiovascular Risk in Diabetes trials, which revealed increased mortality in the intensive treatment group, indicating the potential role of treatment-associated hypoglycemia on adverse cardiovascular outcomes in the T2DM population [27]. Severe hypoglycemia is also known to increase CV risk in the Atherosclerosis Risk in Communities study, which showed a twofold risk of incident or recurrent CVD and a 1.7-fold risk of all-cause mortality [5].

We hypothesized that hypoglycemic events in patients with T2DM might have clinical and prognostic implications in patients hospitalized for HF from the emergency department. Thus, we compared the adverse cardiovascular outcomes and all-cause mortality according to the presence of T2DM and hypoglycemia (reference group: HF without T2DM or hypoglycemia, HF with T2DM without hypoglycemia, HF with T2DM and hypoglycemia) at the time of HF hospitalization and follow-up. In this study, subjects in the group with T2DM and hypoglycemia had higher HbA1c levels than those in the group with T2DM without hypoglycemia (7.8 ± 1.8% vs. 7.3 ± 3.3%, p = 0.004), suggesting that the group with T2DM and hypoglycemia patients might have a higher risk of hypoglycemia with intensified T2DM treatment, which corresponds to ACCORD and the Outcome Reduction With Initial Glargine Intervention trial, which showed severe hypoglycemia in the standard therapy group who experienced a higher relative risk of all-cause mortality [28, 29].

Subjects included in the group with T2DM and hypoglycemia might be in a high-risk state because of the reduced ejection fraction and increased NT-proBNP and high-sensitivity troponin T levels, followed by a high rate of 3P-MACE and increased mortality in HF hospitalization. However, significant associations of hypoglycemic events in HF hospitalization persisted after adjustment for multiple covariates, including SBP, glycemic status, reduced EF, biomarkers, and traditional CV risk factors. Thus, clinicians should pay more attention to avoiding hypoglycemia during HF hospitalization to reduce the risk of increased mortality and adverse CV outcomes.

In this study, a lower body mass index was also associated with an increase in 3P-MACE and all-cause mortality, indicating poor prognosis in HF, which may be associated with poor nutrition or a general unhealthy state that results in weight loss [30].

As in this study, prior studies reported that lower SBP < 100 mm Hg continued to be associated with higher mortality and CV mortality in systolic HF patients [31, 32]. Hypertension often precedes the development of HF and causes mortality in HF. The lower BP in patients with HF may be due to antihypertensive drugs or a state of more advanced HF and low cardiac output [32].

### Potential mechanism of hypoglycemia and T2DM in HF patients

The association between hypoglycemia and T2DM in HF hospitalization may be supported by a potential mechanism. Hypoglycemia is linked to coronary artery calcification in T2DM after modifying glycemic control status, which might be associated with profound surges in the sympathoadrenal system as a counterregulatory mechanism, leading to increased cardiac workload and transient ischemia or cardiac failure. Hypoglycemia is also associated with an increase in proinflammatory markers and cytokines related to endothelial dysfunction, which contributes to atherosclerosis [33, 34].

Hypoglycemia can induce increased myocardial electrical vulnerability and vascular thrombosis [34]. Suggested mechanisms include cardiac arrhythmia, prolongation of cardiac repolarization, and hypoglycemia-associated autonomic failure, which may be associated with lethal ventricular arrhythmia. The association between baseline QTc and CV mortality has been reported in patients with T2DM [35]. Insulin-induced hypoglycemia causes catecholamine, including epinephrine, release and increases the QTc interval [36].

In addition, increased high-sensitivity troponins and natriuretic peptides, including NT-proBNP, are well-known, substantial prognostic biomarkers for acute HF, reflecting subclinical myocardial structural changes, thus providing useful information about underlying disease progression [37]. The group with T2DM and hypoglycemia subjects were in acute HF and showed increased high-sensitivity troponin T and NT-proBNP levels compared with the reference group (those without T2DM or hypoglycemia) or the group with T2DM without hypoglycemia, thus indicating poorer prognoses for cardiovascular outcomes and all-cause mortality. Thus, hypoglycemia and T2DM are associated with all, or a combination, of these mechanisms and could be related to increased mortality and adverse CV outcomes at HF hospitalization.

### Limitations

This study has several limitations. First, this was a retrospective, observational design with a small number of participants. The number of those with T2DM and hypoglycemia was small, and the baseline characteristics of participants were discordant according to the presence of hypoglycemia and T2DM. This study might have had the remaining unmeasured confounding variables leading to biased results. Thus, we could not control for all confounding factors influencing CV outcomes and mortality, although we tried to minimize this effect by adjusting for multiple conventional risk factors. In addition, fasting plasma glucose, HbA1c, renal function, and cardiac biomarkers were confirmed only once, and we could not evaluate the serial changes after HF hospitalization or the effect of changes in variables on incident MACE effects. Further prospective studies are needed to confirm the causal relationship between hypoglycemia and adverse CV outcomes in patients with HF.

Second, we only have data on the Korean population.

## Conclusion

This study suggests that hypoglycemia in T2DM is an independent risk factor for 3P-MACE and all-cause mortality compared to those without hypoglycemia in HF hospitalization from the emergency department. The relationship between hypoglycemia in T2DM and cardiovascular outcome was independent of traditional CV risk factors and the value of several biomarkers, including high-sensitivity troponin T, NT-proBNP, and C-reactive protein. Clinicians should pay more attention to preventing and reducing the risk of hypoglycemia in hospitalized patients with HF. Further studies are needed to investigate the pathogenic mechanism of hypoglycemia for adverse CV outcomes and increased mortality in HF patients.

## Supplementary Information

**Additional file 1: Table S1.** Baseline characteristics according to the presence of hypoglycemia in patients with T2DM and heart failure. **Table S2.** Adverse cardiovascular outcome and all-cause mortality according to group. **Table S3.** Multivariable Cox hazard regression model for the adverse cardiovascular outcome and all-cause mortality in patients with heart failure.

## Data Availability

The data of this study may be available on reasonable request to the corresponding author.

## Abbreviations

T2DM: Type 2 diabetes
HF: Heart failure
MI: Myocardial infarction
CV: Cardiovascular
eGFR: Estimated glomerular filtration rate
NT-pro BNP: N-terminal-pro-B-type natriuretic peptide
CKD: Chronic kidney disease
3P-MACE: Three-point major adverse cardiovascular events
CVD: Cardiovascular disease
SBP: Systolic blood pressure
