# Supplementary material for: Impact of hypoglycemia at the time of hospitalization for heart failure from emergency department on major adverse cardiovascular events in patients with and without type 2 diabetes
Source: Cardiovasc Diabetol. 2022 Oct 21;21:218. doi: 10.1186/s12933-022-01651-0 (PMC9585717; doi:10.1186/s12933-022-01651-0)
Supplement: Supplementary file 1 — Additional file 1: Table S1. Baseline characteristics according to the presence of hypoglycemia in patients with T2DM and heart failure. Table S2. Adverse cardiovascular outcome and all-cause mortality according to group. Table S3. Multivariable Cox hazard regression model for the adverse cardiovascular outcome and all-cause mortality in patients with heart failure. [file 12933_2022_1651_MOESM1_ESM.docx]

**Table S1. Baseline characteristics according to the presence of hypoglycemia in patients with T2DM and heart failure.**

|  | **Heart failure**  **with T2DM without hypoglycemia** | **Heart failure with T2DM, hypoglycemia** | ***P* value** |
| --- | --- | --- | --- |
| n (total =397) | 318 (40.6) | 79 (10.1) |  |
| Age (years) | 76.0 (66.8–81.0) | 76.0 (67.0–83.0) | 0.654 |
| Male | 147 (46.2) | 41 (51.9) | 0.366 |
| Body mass index (kg/m^2^) | 24.0 (22.1–26.6) | 23.5 (20.8–26.3) | 0.079 |
| Smoking |  |  | 0.292 |
| Current | 48 (15.1) | 8 (10.1) |  |
| Ex-smoker | 36 (11.3) | 13 (16.4) |  |
| T2DM | 318 (100.0) | 79 (100.0) | - |
| Duration of T2DM (years) | 11.2 ± 10.5 | 14.5 ± 11.7 | 0.044 |
| Hypertension | 239 (75.2) | 62 (78.5) | 0.537 |
| History of CHD | 71 (22.3) | 24 (30.4) | 0.133 |
| History of stroke | 35 (11.0) | 9 (11.4) | 0.922 |
| Previous HF | 58 (18.2) | 20 (25.3) | 0.156 |
| Duration of HF (years) | 6.1 ± 5.4 | 3.8 ± 3.1 | 0.176 |
| Etiology of HF |  |  | 0.162 |
| Ischemic cause (coronary artery disease or myocardial infarction) | 110 (34.6) | 34 (43.0) |  |
| Nonischemic cause | 208 (65.4) | 45 (57.0) |  |
| Chronic kidney disease | 166 (52.2) | 54 (68.4) | 0.013 |
| Systolic blood pressure (mm Hg) | 135.0 ± 28.8 | 135.1 ± 26.4 | 0.975 |
| Diastolic blood pressure (mm Hg) | 80.3 ± 16.7 | 77.8 ± 13.6 | 0.231 |
| Heart rate (beats per min) | 92.8 ± 21.9 | 93.7 ± 23.3 | 0.742 |
| Left ventricular ejection fraction (%) | 43.5 ± 15.0 | 38.7 ± 14.6 | 0.013 |
| E/e’ | 21.0 ± 10.2 | 20.9 ± 9.0 | 0.964 |
| **Laboratory variables** |  |  |  |
| FPG (mmol/L) | 8.52 ± 4.1 | 3.3 ± 0.7 | < 0.001 |
| HbA1c (%) | 7.3 ± 3.3 | 7.8 ± 1.8 | 0.004 |
| eGFR (mL•min^-1^•1.73m^-2^) | 57.1 (36.5–83.8) | 45.9 (29.4–69.2) | 0.009 |
| Total cholesterol (mmol/L) | 3.8 ± 1.1 | 3.5 ± 1.2 | 0.034 |
| Triglyceride (mmol/L) | 1.0 (0.8–1.4) | 0.8 (0.7–1.1) | 0.013 |
| HDL-C (mmol/L) | 1.1 ± 0.4 | 1.1 ± 0.3 | 0.950 |
| LDL-C (mmol/L) | 2.4 ± 0.9 | 2.2 ± 0.9 | 0.083 |
| NT-pro BNP (pg/mL) | 4250 (1828–9598) | 7485 (3266–24153) | < 0.001 |
| HsTnT (ng/L) | 32.0 (18.0–75.5) | 66.0 (27.0–133.3) | 0.001 |
| CK-MB (ng/ml) | 3.1 (2.1–5.5) | 4.1 (2.4–6.3) | 0.049 |
| CPK (U/L) | 103.0 (66.0–166.5) | 104.0 (62.0–164.0) | 0.961 |
| CRP (mg/dl) | 0.5 (0.2–2.1) | 1.0 (0.2–3.2) | 0.088 |
| **Medication** |  |  |  |
| **Cardiovascular medication** |  |  |  |
| ACEi/ARB | 122 (38.4) | 29 (36.7) | 0.786 |
| Beta blocker | 98 (30.9) | 25 (31.6) | 0.900 |
| CCB | 96 (30.4) | 28 (35.4) | 0.386 |
| Diuretics | 143 (45.0) | 45 (57.0) | 0.056 |
| Aspirin | 87 (27.4) | 26 (32.9) | 0.328 |
| Statin | 106 (33.3) | 29 (36.7) | 0.571 |
| **Diabetes treatment** |  |  |  |
| Insulin | 29 (9.1) | 11 (13.9) | 0.204 |
| Sulfonylurea | 59 (18.6) ^#^ | 30 (38.0) ^#^ | < 0.001 |
| Metformin | 94 (29.6) | 26 (32.9) | 0.562 |
| DPP-4 inhibitor | 99 (31.1) | 28 (35.4) | 0.462 |

Data are number (percentage) or medians with 25th–75th percentiles, means ± SD.

CHD coronary heart disease, BMI body mass index, T2DM, type 2 diabetes, SBP systolic blood pressure, DBP diastolic blood pressure, FPG fasting plasma glucose, eGFR estimated glomerular filtration rate, HDL high-density lipoprotein, LDL low-density lipoprotein, NT-proBNP N-terminal-pro-B-type natriuretic peptide, hsTnT High-sensitivity troponin T, ACEi/ARB, ACE inhibitor/angiotensin receptor blocker, CCB, calcium channel blocker

**Table S2. Adverse cardiovascular outcome and all-cause mortality according to group.**

|  | **Heart failure**  **without T2 DM**  **(N = 386)** | **Heart failure**  **with T2DM without hypoglycemia**  **(N = 318)** | **Heart failure with T2DM, hypoglycemia**  **(N = 79)** | ***P* for trend** |
| --- | --- | --- | --- | --- |
| 3P-MACE | 57 (14.8) | 71 (22.3) | 31 (39.2) | < 0.001 |
| Nonfatal MI | 9 (2.3) | 19 (6.0) | 6 (7.6) | 0.006 |
| Nonfatal stroke | 11 (2.8) | 15 (4.7) | 3 (3.8) | 0.337 |
| Cardiovascular mortality | 37 (9.6) | 37 (11.6) | 22 (27.8) | < 0.001 |
|  |  |  |  |  |
| All-cause mortality | 55 (14.2) | 69 (21.7) | 34 (43.0) | < 0.001 |

Data are number (percentage). *P* < 0.05 was considered significant.

**Table S3. Multivariable Cox hazard regression model for the adverse cardiovascular outcome and all-cause mortality in patients with heart failure.**

|  | **Incident 3P-MACE** | | **Cardiovascular mortality** | | **All-cause mortality** | |
| --- | --- | --- | --- | --- | --- | --- |
|  | **Adjusted** | | **Adjusted** | | **Adjusted** | |
|  | **Hazard ratio (95% CI)** | ***P* value** | **Hazard ratio (95% CI)** | ***P* value** | **Hazard ratio (95% CI)** | ***P* value** |
| **Age (per 10 years)** | 1.00 (0.98–1.02) | 0.980 | 1.01 (0.99–1.03) | 0.314 | 1.02 (1.00–1.04) | 0.022 |
| **Sex (male)** | 1.07 (0.71–1.61) | 0.749 | 1.09 (0.67–1.79) | 0.725 | 0.92 (0.62–1.37) | 0.691 |
| **Log (body mass index [kg/m^2]^)** | 0.19 (0.06–0.62) | 0.006 | 0.12 (0.03–0.49) | 0.003 | 0.13 (0.04–0.41) | < 0.001 |
| **Current smoking status (yes vs. no)** | 1.10 (0.64–1.89) | 0.739 | 0.75 (0.36–1.56) | 0.435 | 0.89 (0.48–1.63) | 0.697 |
| **History of CHD (yes vs. no)** | 0.77 (0.39–1.52) | 0.447 | 1.17 (0.49–2.81) | 0.719 | 1.06 (0.53–2.14) | 0.863 |
| **History of stroke (yes vs. no)** | 1.02 (0.56–1.85) | 0.950 | 0.74 (0.32–1.71) | 0.483 | 0.72 (0.38–1.38) | 0.320 |
| **History of heart failure (yes vs. no)** | 1.89 (1.18–3.03) | 0.008 | 2.29 (1.31–3.99) | 0.004 | 1.68 (1.07–2.63) | 0.023 |
| **Etiology of heart failure (ischemic vs. nonischemic)** | 1.68 (0.94–3.01) | 0.082 | 1.15 (0.54–2.49) | 0.721 | 1.17 (0.63–2.19) | 0.620 |
| **Diabetes duration (≥ 10 years) (yes vs. no)** | 0.80 (0.47–1.34) | 0.394 | 0.60 (0.30–1.18) | 0.139 | 0.57 (0.34–0.96) | 0.034 |
| **Systolic blood pressure (per 10 mmHg)** | 0.88 (0.82–0.95) | 0.001 | 0.89 (0.81–0.98) | 0.020 | 0.91 (0.85–0.98) | 0.012 |
| **Log (fasting plasma glucose [mmol/L])** | 1.23 (0.66–2.29) | 0.511 | 1.58 (0.72–3.48) | 0.257 | 1.42 (0.77–2.65) | 0.264 |
| **HbA1c ≤ 7.0% (yes vs. no)** | 0.71 (0.49–1.03) | 0.073 | 0.70 (0.44–1.11) | 0.125 | 0.76 (0.53–1.10) | 0.146 |
| **eGFR ≤ 60 mL/min/1.73m^2^ (yes vs. no)** | 0.79 (0.52–1.21) | 0.282 | 0.63 (0.37–1.08) | 0.091 | 0.90 (0.59–1.37) | 0.612 |
| **Ejection fraction ≤ 40% (yes vs. no)** | 1.11 (0.75–1.63) | 0.615 | 1.21 (0.74–1.98) | 0.445 | 1.05 (0.71–1.53) | 0.818 |
| **Insulin use (yes vs. no)** | 1.15 (0.51–2.55) | 0.741 | 1.17 (0.39–3.51) | 0.779 | 1.77 (0.88–3.58) | 0.109 |
| **Sulfonylurea use (yes vs. no)** | 1.13 (0.63–2.02) | 0.690 | 1.22 (0.61–2.46) | 0.576 | 1.18 (0.69–2.02) | 0.547 |
| **Antihypertensive medication use (yes vs. no)** | 1.30 (0.84–2.02) | 0.240 | 1.06 (0.62–1.81) | 0.834 | 1.41 (0.90–2.20) | 0.130 |
| **Statin use (yes vs. no)** | 0.74 (0.47–1.15) | 0.174 | 0.73 (0.41–1.28) | 0.265 | 0.85 (0.56–1.29) | 0.438 |
| **Log (hsTnT [ng/L])** | 1.09 (0.94–1.27) | 0.259 | 1.19(0.99–1.43) | 0.070 | 1.19 (1.02–1.38) | 0.025 |
| **Log (NT-pro BNP [pg/mL])** | 1.28 (1.06–1.55) | 0.010 | 1.55 (1.20–2.01) | 0.001 | 1.37 (1.13–1.66) | 0.002 |
| **Log (CRP [mg/dl])** | 1.07 (0.95–1.21) | 0.290 | 1.06 (0.92–1.23) | 0.077 | 1.08 (0.96–1.21) | 0.190 |
| **Group** |  |  |  |  |  |  |
| **Patients without T2DM (ref.)** | 1.00 |  | 1.00 |  | 1.00 |  |
| **Patients with T2DM** | 1.42 (0.86–2.33) | 0.169 | 1.12 (0.61–2.07) | 0.528 | 1.32 (0.81–2.16) | 0.259 |
| **Patients with T2DM and hypoglycemia** | 2.29 (1.04–5.06) | 0.040 | 2.88 (1.17–7.05) | 0.021 | 2.58 (1.26–5.31) | 0.010 |

3P-MACE three-point major adverse cardiovascular events, CI confidence interval, CHD coronary heart disease, hsTnT High-sensitivity troponin T, NT-proBNP N-terminal-pro-B-type natriuretic peptide, CRP C-reactive protein.
